# Apheresis Therapy for Steroid-Resistant Idiopathic Nephrotic Syndrome: Report on a Case Series

**DOI:** 10.1155/2019/7304786

**Published:** 2019-10-09

**Authors:** Hamza Naciri Bennani, Thomas Jouve, Johan Noble, Lionel Rostaing, Paolo Malvezzi, Rachel Tetaz

**Affiliations:** ^1^Service de Néphrologie, Hémodialyse, Aphérèses et Transplantation Rénale, CHU Grenoble-Alpes, Grenoble, France; ^2^Université Grenoble Alpes, Grenoble, France

## Abstract

Idiopathic nephrotic syndrome (INS) represents 15%–30% of adulthood glomerulopathies. Corticosteroids have been the main treatment for decades and are effective in 70% of minimal-change disease patients and ~30% of focal segmental glomerulosclerosis patients. Multidrug-resistant (steroids, calcineurin-inhibitors, cyclophosphamide, mycophenolate-mofetil, rituximab) idiopathic nephrotic syndrome is a major therapeutic challenge in nephrology. Apheresis (double-filtration plasmapheresis or semi specific immunoadsorption) could act by eliminating the circulating factor (apolipoproteinA1b, solubleCD40L, suPAR) increasing glomerular permeability seen in INS. The aim of the study was to report the outcome of three patients with multidrug-resistant INS treated successfully with apheresis.

## 1. Introduction

Idiopathic nephrotic syndrome (INS) causes ~90% of childhood glomerulopathies and between 15%–30% of adulthood glomerulopathies [1]. INS is the result of either minimal-change disease (MCD) or focal-segmental glomerulosclerosis (FSGS). In adult patients, first-line treatment relies on steroids, leading to full-remission in 70% of MCD patients [2] and ~30% of FSGS patients [3]. Complete remission of proteinuria with prednisone is a major prognostic factor. In cases of steroid-dependence or steroid-resistant INS, second-line therapy relies on calcineurin-inhibitors, cyclophosphamide, mycophenolate-mofetil (MMF), or rituximab [4]. In ~50% of cases, steroid-resistant INS evolves to end-stage kidney disease within 6–8 years. When INS does not respond to immunosuppressive drugs, apheresis can be considered (e.g., double-filtration plasmapheresis (DFPP) or semi specific immunoadsorption (IA)).

DFPP (plasma separator Plasmaflo™ OP-056 W; Cascadeflo™ EC20; Asahi Kasei Medical, Tokyo, Japan) is an apheresis technique that removes high molecular-weight proteins (alpha2-macroglobulin, LDL, fibrinogen, and immunoglobulins mainly IgM). The blood passes through the first filter, which is used to separate cellular components consisting of white blood cells, red blood cells, and platelets from the plasma. The plasma without the cellular components will pass through the second filter where the macromolecules are selectively removed. Semi-specific immunoadsorption (Immunosorba® or Globaffin® columns; Fresenius Medical Care, Bad-Homburg, Germany) initially involves a centrifugation separating the plasma and cellular components. The plasma is then treated with an adsorptive membrane to selectively remove immunoglobulins (IgA, IgM, and IgG).

Several reports of the literature involve antibodies or circulating factor with regard to the mechanism of proteinuria in INS that triggers podocytes causing the increased glomerular permeability (e.g., apolipoproteinA1b, solubleCD40L, suPAR) [5]. These antibodies and/or this circulating factor would also be the cause of recurrence INS on the kidney allograft [5]. Apheresis could act by eliminating the antibodies and the circulating factor.

Full remission is defined as having proteinuria <0.5 g/day and albuminemia >30 g/L; partial remission is defined as proteinuria >0.5 g/day, and albuminemia >30  g/L, or a 50% reduction in the initial proteinuria [6].

**Figure 1:**
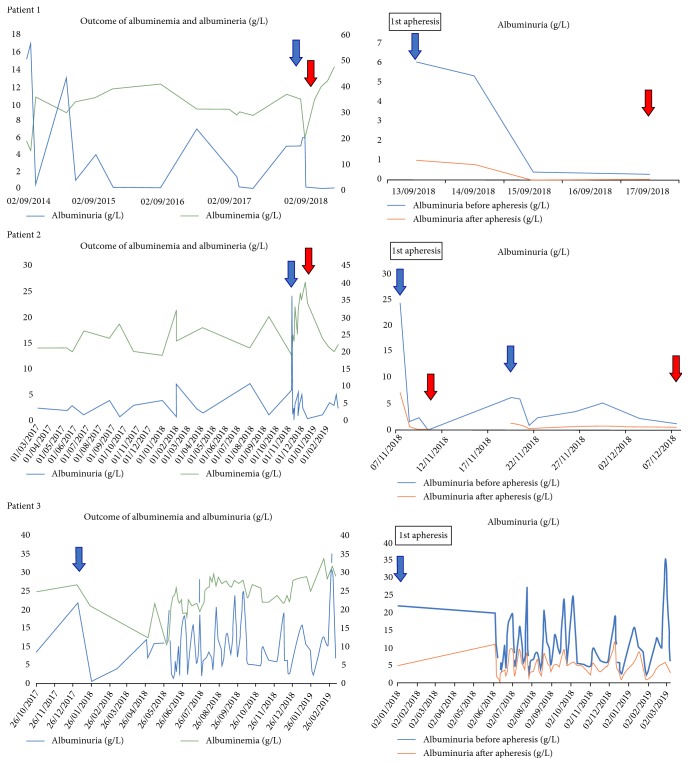
Outcome of albuminemia and albuminuria (g/L) for three patients. The blue arrow corresponds to the beginning of apheresis therapy and the red arrow corresponds to the stop of apheresis therapy. On the x axis are dates of the sessions and y axis on the left side albuminuria (g/L) and on the right side albuminemia (g/L).

Herein, we report on three adult patients with immunosuppressive-resistant INS and that they were successfully treated with apheresis (Figure 1).

## 2. Case Series

### 2.1. Patient 1

A 66-year-old male was diagnosed in 2014 with MCD (proteinuria 15 g/L/ albuminemia 19 g/L). He was initially treated with steroids, which gave a good response, but he became steroid-dependent (20 mg/d). When corticosteroid therapy was decreased (<20 mg/d), proteinuria increased to 13 g/L. He, thereafter, received four infusions of rituximab (1 gr each) over a 3-year period, with low steroid doses. The first 3 infusions of rituximab allowed a complete remission during, respectively, 3, 1, and 8 months: at the time of each relapse proteinuria was found to be at 4, 7, and 5 g/L, respectively. At the time of the fourth relapse he received an infusion of rituximab (the 4th one); this induced partial remission; however, one month later proteinuria reincreased to 6 g/L.

In September 2018, he had a fifth relapse (proteinuria 6 g/L/albuminemia 28 g/L). At that point, he received DFPP (one daily session for 4 consecutive days) followed by one infusion of rituximab (1 gr), and subsequently, remission (>10 months) (>6 months) with proteinuria at 0.09 g/L and albuminemia at 46 g/L. He was weaned-off steroids at the end of DFPP sessions. His renal function remained normal (i.e., estimated glomerular-filtration rate (eGFR) at 89 mL/min/1.73 m^2^ according to CKD-EPI formula).

### 2.2. Patient 2

A 44-year-old woman was diagnosed with MCD when aged 13. She achieved remission with steroids. During her second pregnancy in 2016, she had a relapse of MCD (proteinuria 2.5 g/L/albuminemia 21 g/L) and was placed on steroid therapy without success. Therefore, from early 2017 to November 2018, she was successively treated with MMF, rituximab, and tacrolimus without success. A second kidney biopsy confirmed MCD.

In November 2018, proteinuria was 6 g/L and albuminemia 19 g/L under tacrolimus. She was started on IA therapy, i.e., one daily session for 4 days, which induced remission (proteinuria 0.26 g/L albuminemia 23 g/L). After one week without IA, proteinuria reappeared (proteinuria 5 g/L, and albuminemia 25 g/L); thus, IA therapy was resumed (i.e., one session/day 4 days, then two sessions per week for two weeks). This resulted in partial remission (proteinuria 1.6 g/L albuminemia 40 g/L). However, she refused to continue IA therapy, corticoids, and tacrolimus. Currently, proteinuria fluctuates between 2 and 5 g/L, and albuminemia is between 20 and 25 g/L. Renal function is normal (i.e., eGFR is 106 mL/min/1.73 m^2^).

### 2.3. Patient 3

A 39-year-old male was diagnosed in October 2017 with MCD; proteinuria was 8.8 g/L and albuminemia was 25 g/L. Partial remission was achieved with steroids. Tacrolimus was then added, but was stopped after 3 months because of no improvement in proteinuria (proteinuria 12 g/L albuminemia 13 g/L). In June 2018, apheresis was started, alternating DFPP (up to 18) and IA (up to 36 sessions). In addition, he received an infusion of rituximab (1 gr) every 3 months, and MMF was added. We were unable to stop apheresis because of a relapse within 8–10 days. At present, he receives one apheresis session/week and is no longer taking immunosuppressive therapy; proteinuria fluctuates between 3 and 10 g/L, and albuminemia between 25 and 35 g/L. His eGFR is 79 mL/min/1.73m^2^.

To summarize, these three cases of immunosuppressive-resistant INS that received apheresis allowed (i) full remission in one case (with steroid withdrawal), (ii) partial remission in one case with apheresis dependency (one session/week); and (iii) partial remission with IA dependency in the third case, but the patient refused long-term therapy.

## 3. Discussion

The physiopathology of FSGS is still unknown and relies on the existence of a permeability circulating factor (suPAR) or antibodies (anti-CD40 antibody, protein tyrosine-phosphatase receptor type O antibody, apoliprotein,…) [5]. A recent study identified anti-ubiquitin carboxyl-terminal hydrolase 1 autoantibody (UCHL-1) as a cause of proteinuria in INS. This antibody induces proteinuria and podocyte foot effacement in mice [7]. If autoantibody (ies) is/are involved in the pathophysiology of INS, rituximab and apheresis may be the best therapy for corticosteroid-resistant INS. Rituximab is a B-lymphocyte depleting monoclonal antibody that targets CD20. It can influence the production of the circulating factor that results in INS and it inhibits actin-cytoskeleton breakdown in podocytes to reduce proteinuria [8]. On the other hand, apheresis could act by directly eliminating the antibodies and the circulating factor.

In a review paper, Muso et al. reported that apheresis resulted in 54%–86% remission in adults with steroid-resistant INS [9]. In a prospective multicenter study that included 44 steroid/cyclosporine-resistant adults with INS (12 MCD and 28 FSGS) apheresis achieved partial remission in 23% after 9.6+/−2.7 sessions [10] and was 84% more efficacious when started earlier (i.e. at <8 weeks vs. later [43% success]).

Raina et al. reported on 11 steroid/cyclosporine-resistant children with INS where apheresis induced remission in 64%, i.e., proteinuria decreased from 9.7+/−2.5 to 1 +/0.5 g/d [11]. Similarly, Nattes et al. [12] gave IA (10 sessions over a 2-week period) to 14 steroid/calcineurin-inhibitor-resistant children with INS (58% were FSGS); this resulted in full or partial remission in 50% and 15% of cases, respectively.

## 4. Conclusion

Until now, there has been no consensus on when to implement apheresis in immunosuppressive-resistant INS (in both children and adults). However, the literature and our experience suggest that DFPP or IA can be added in INS when conventional therapies have failed.
